# Metabolomics and Transcriptomics Reveal Age-Dependent Development of Meat Quality Traits in Jingyuan Chicken

**DOI:** 10.3390/ani15131938

**Published:** 2025-07-01

**Authors:** Jiahuan Hu, Wei Zhao, Jinyan Zhao, Jinli Tian, Lijuan Yang, Hua Wang, Siyu Chen, Ruimin Ma, Yaling Gu, Dawei Wei, Juan Zhang

**Affiliations:** College of Animal Science and Technology, Ningxia University, Yinchuan 750000, China; hujiahuan2022@163.com (J.H.); z1729921403w@163.com (W.Z.); 15709616397@163.com (J.Z.); anti@163.com (J.T.); yangdfq@163.com (L.Y.); willa.mmar520@gmail.com (H.W.); 13239608379@163.com (S.C.); 15039057688@163.com (R.M.); guyaling@sina.com (Y.G.)

**Keywords:** Jingyuan chickens, metabolome, transcriptome, meat quality traits, age

## Abstract

In this study, we investigated the dynamic changes in muscle lipid content (MLC) deposition in the breast muscle of Jingyuan chickens at different stages (42-, 126-, and 180- days old) using metabolomics. The pH_45min,_ a*, and L* were significantly increased in the breast muscle of 126-day-old chickens, and MLC and b* were significantly increased in the breast muscle of 180-day-old chickens. A total of 4643 differentially expressed metabolites (DEMs) were identified, of which 10 decreased and 29 increased with age. Key pathways associated with meat quality traits included oxidative phosphorylation, β-alanine metabolism, and glycerophospholipid metabolism. Combined transcriptomic and phenotypic correlation analyses revealed significant positive correlations of MLC, pH_45min_, a*, and L* with LysoPC 20:4, *CD3E*, *TARP*, *IL7R*, *ENSGALG00010025331*, and *RASSF5*. In the 42- and 180-day-old chickens, MLC, pH_45min_, a*, and L* were significantly and positively correlated with L-Anserine, Dihydroxyacetone phosphate, *ENSGALG00010006904*, and *HSPB7*. MLC, pH_45min_, a*, and L* were significantly and positively correlated with beta-Nicotinamide adenine dinucleotide in the 126- and 180-day-old chickens. This study deepens our understanding of the differences in MLC deposition at different stages of Jingyuan chickens and its relationship with meat quality traits.

## Simple Summary

This study explored how muscle lipid content (MLC) deposition and meat quality traits change at different stages of development in Jingyuan chickens. We analyzed the breast muscle of 42-, 126- and 180-day-old chickens using metabolomics techniques. Our findings showed that certain meat quality traits, such as pH_45min_, color values (a* and L*), and MLC, varied significantly with age. We identified 4643 differentially expressed metabolites (DEMs), some of which increased while others decreased as the chickens aged. Key metabolic pathways related to meat quality traits included oxidative phosphorylation, β-alanine metabolism, and glycerophospholipid metabolism. We also found strong correlations between MLC, pH_45min_, and color traits with specific metabolites and genes, providing a deeper understanding of how fat deposition affects meat quality traits at different growth stages. This research offers valuable insights for improving meat quality traits in Jingyuan chickens.

## 1. Introduction

The only animal product whose price and demand have kept rising is chicken, according to the International Market Commodity Prices 2023 [1]. Production of chicken meat is increasing due to changes in consumer preferences and demand. Although the production of chicken meat has been rising annually in China, where it is the second most consumed meat product, its export volume is less than that in the US and Brazil [2]. Thus, producing superior local chicken breeds with exceptional qualities has become a key area of research in contemporary livestock and poultry molecular breeding in an effort to boost competitiveness and enhance meat flavor [3].

Muscle lipid content (MLC) is one of the important indexes for evaluating meat quality traits. Meat flavor, softness, and food value are all strongly connected with intramuscular fat levels [4]. There are two primary reasons why intramuscular fat improves meat quality traits. First, myofibrillar bundles are dissolved by the oxidation of MLC, improving muscle juiciness and tenderness [5]. Second, when MLC degrades, it can release a range of volatile aromatic chemicals that are essential for enhancing the flavor of meat [6]. Through processes like oxidative lipid degradation, Maillard reaction, thermal amino acid degradation, and thiamine degradation, flavor precursors in chicken meat generate volatile flavor substances, primarily including ketones, aldehydes, esters, alcohols, phenols, sulfur-containing straight-chain compounds, and heterocyclic compounds [7]. One of the five district-level livestock and poultry genetic resources protection breeds of the Ningxia Hui Autonomous Region is Jingyuan chicken, a superior local resource [1]. Because of their distinctive geographic location and rearing practices, Jingyuan chickens are the first choice for “green nutritional and health care” chicken food because of their high nutritional value, low cholesterol, rich fatty and amino acids, and delicious meat [8]. They are also a perfect animal model for research on MLC deposition in muscle [3].

With the rapid growth of science and technology, metabolomics and transcriptomics sequencing technologies are increasingly applied in genetic breeding research on cattle and poultry [9]. Metabolomic analysis of metabolites and lipid pathways in Chaffinch 2 and Yao Chicken revealed multiple differentially expressed metabolites (DEMs) and many metabolic pathways related to amino acids and fatty acids in eight categories [10]. Additionally, the metabolomic analysis of serum and breast muscle in Chaffinch 2 and Yao Chicken showed that changes in myo-glycogen content in chicken meat were closely related to the final pH of the meat, enabling accurate prediction of chicken meat quality traits [11]. Meat metabolites are synthesized and accumulated through genetic regulation [12]. The transcriptomic analysis of Nandap scallop chickens with high and low abdominal fat rates identified *PLA2G4A* and *RPS4Y1* as potential key regulators of fat deposition [13]. Similarly, transcriptomic analysis of Jingxing yellow chickens with high and low triglyceride content revealed that five genes (*DHCR24*, *LSS*, *MSMO1*, *NSDHL*, and *CH25H*) are involved in steroid biosynthesis, while nine genes (*ADIPOQ*, *CD36*, *FABP4*, *FABP5*, *LPL*, *SCD*, *PLIN1*, *CIDEC*, and *PPARG*) are associated with adipogenesis [14]. Furthermore, the primary pathways controlling variations in lipid deposition between high and low triglyceride groups were the steroid biosynthesis and PPAR signaling pathways, which are linked to lipid storage and metabolism. In contrast, pathways related to muscle MLC deposition in the breast and hamstring muscles of Jingyuan chickens, as identified through transcriptomic sequencing, include purine metabolism, glycolysis, pyruvate metabolism, and amino acid biosynthesis [15].

Although past research has explored the mechanism of chicken meat flavor using transcriptomics and metabolomics, the dynamic changes affecting MLC deposition in chicken meat remain less understood. Therefore, we aimed to investigate the molecular mechanisms underlying meat quality traits and flavor during the development of Jingyuan chickens, using both transcriptomic and non-targeted metabolomic approaches. This study examines the DEGs and DEMs affecting MLC deposition in the muscles of Jingyuan chickens at various stages of development.

## 2. Materials and Methods

### 2.1. Ethical Statement

All our experimental procedures were in accordance with the Guidelines for Experimental Animals established by the Ministry of Science and Technology of the People’s Republic of China which were approved by the Animal Care Committee of Ningxia University under ethical approval number NXU-2024-067.

### 2.2. Animal and Sample Collection

A total of 300 1-day-old Jingyuan chicks were reared at the Jingyuan Chicken National Breeding Farm (Panyang County, Guyuan, China) using standardized temperature and humidity. All hens were always fed and watered ad libitum under the same conditions. Since 42 days of age is the beginning of the breeding period of Jingyuan chickens, 126 days is the point of sexual maturity of Jingyuan chickens, and their weight stabilizes at 180 days, which is the typical market age, we chose 42-, 126-, and 180-day-old chickens to explore the changes in MLC deposition during the growth process of chickens. Group B1 was slaughtered at 42 days (0.422 ± 0.03 kg), Group B2 was slaughtered at 126 days (1.68 ± 0.14 kg), and Group B3 was slaughtered at 180 days (2.03 ± 0.09 kg). After rendering the hens unconscious with an ether respiratory anesthetic, we severed their carotid arteries and ended their lives. Thirty hens were randomly selected from each group, whole muscles were collected for meat properties, and the remaining breast muscle was snap-frozen in liquid nitrogen and stored at −80 °C in a refrigerator. Finally, 10 hens of the same weight were selected from each age group and their breast muscles were sequenced for the transcriptome and untargeted metabolome. The sample numbers are shown in Table S1.

### 2.3. Meat Quality Traits Determination

Using a pH meter (PH-STAR, Meters, Denmark), the pH of the meat was measured 45 min after slaughter at three distinct sites on the upper, middle, and bottom portion of each breast muscle, each 10 mm deep. Prior to usage, the pH meter was calibrated twice using reference buffers with pH values of 4.0 and 7.0 to guarantee the accuracy of the readings. A spectrophotometer 968 (CR-400, Minolta, Japan) that had been calibrated in black and white prior to usage was used to measure the color parameters of the breast muscle. Each breast muscle’s L*, a*, and b* values were measured three times, and the mean value was used to determine the final meat color. In accordance with the method specified in GB 5009.6-2016 [16] for the determination of fat in food, we employed the first Soxhlet extractor method. First, we carefully weighed 5 g of the well-mixed sample of the breast muscle, accurate to the nearest 0.001 g. This weighed sample was then placed entirely into a filter cylinder. Next, the filter paper cylinder containing the sample was placed in the extraction chamber of the Soxhlet extraction apparatus. Meanwhile, we ensured that a receiver flask, which had been dried to a constant weight beforehand, was prepared. The extraction apparatus was then set up by combining the extraction chamber with the receiver flask to initiate the extraction process for determining the MLC. Petroleum ether was added to the flask through the upper end of the condenser tube, filling it up to two-thirds of its volume. The apparatus was then heated in a water bath, and continuous reflux extraction was performed using anhydrous ethyl ether or petroleum ether at a rate of 6 to 8 cycles per hour, typically for a period of 6 to 10 h. Once the extraction was complete, the receiver flask was removed, the anhydrous ether or petroleum ether were recovered, and the remaining 1 to 2 mL of solvent was evaporated in a water bath. The solvent was then dried at 100 °C ± 5 °C for 1 h, followed by cooling in a desiccator for 0.5 h before weighing. This process was repeated until a constant weight was achieved (when the difference between two consecutive rounds of weighing was less than 2 mg). The fat content of the sample was then calculated using the following formula: [Content of receiving flask and fat after constant weight (g)−mass of receiving flask (g)] / mass of sample × 100%.

### 2.4. Metabolomics Analysis by Untargeted HPLC-HRMS

After thawing the breast muscle samples on ice, 80% methanol buffer was used to extract the metabolites. In short, 0.5 mL of 80% methanol that had been chilled beforehand was used to extract 50 mg of breast muscle. At −20 °C, the extraction mixture was left to stand for 30 min. Following a 15 min centrifugation at 20,000× *g*, the supernatant was moved to a fresh tube and vacuum-dried. After being redissolved in 100 μL of 80% methanol, the samples were subjected to HPLC-HRMS analysis. Additionally, pooled QC samples were prepared by mixing 10 μL of each extraction mixture [17]. Thermo Scientific UltiMate 3000 HPLC (Thermo Scientific, Waltham, MA, USA) with an ACQUITY UPLC BEH C18 (Waters, Milford, MA, USA) column was employed as an ultra-high-performance liquid chromatography system. The mobile phases for chromatographic acquisition were acetonitrile (0.1% formic acid) in phase B and water (0.1% formic acid) in phase A. The gradients in the liquid phase were established as follows: 0–0.5 min, 5% B; 0.5–7 min, 5–100% B; 7–8 min, 100% B; 8–8.1 min, 100–5% B; 8.1–10 min, 5% B. A Q-Exactive (Thermo Scientific, MA, USA), high-resolution tandem mass spectrometer was then utilized for the column containing the eluted metabolites. Both positive and negative ion modes were used by Q-Exactive. To reach an AGC objective of 3e6, precursor spectra were gathered at 70,000 resolution (70–1050 *m*/*z*). One hundred milliseconds was the maximum injection time. The initial 3-bit set up was configured to use DDA mode for data acquisition. To assess the stability of the HPLC-HRMS throughout the collection process, a quality traits control sample (a collection of all samples) was collected after every 10 samples.

Following the acquisition of the downstream data, XCMS 4.7 software was used to preprocess the mass spectrometry data [18]. The mzXML format was used to transform the HPLC-HRMS raw data files. Metabolites were annotated using ion intensity data, peak intensities, and sample observations. The metabolites were annotated by comparing the data in the Human Metabolome Database (HMDB, https://hmdb.ca/) and Kyoto Encyclopedia of Genes and Genomes (KEGG, http://www.genome.jp/kegg/) (accessed on the 9 of November 2024) using precise molecular mass data (*m*/*z*). Using principal component analysis (PCA) and orthogonal least partial squares discriminant analysis (OPLS-DA), the metaX program was utilized to evaluate the extent of sample differences. Fold Change (FC) > 1.5 or FC ≤ 1/1.5, VIP ≥ 1, and *p* < 0.05 were used to select DEMs. The identified DEMs were subjected to advanced Mfuzz analysis using the OmicStudio tool on https://www.omicstudio.cn/tool (accessed on the 16 November 2024).

### 2.5. Transcriptomics Analysis

Transcriptome results from our previous data [19]. On an Illumina NovaseqTM 6000 (LC-Bio Technology CO., Ltd., Hangzhou, China), we carried out 2 × 150 bp paired-end sequencing (PE150) in accordance with the supplier’s suggested procedure. Trizol reagent (Thermofisher, MA, USA) was used to extract total RNA from breast muscle samples in accordance with the manufacturer’s instructions. A Bioanalyzer 2100 and RNA 6000 Nano LabChip Kit (Agilent, Santa Clara, CA, USA) was used to measure the concentration and purity of total RNA. Thermo Fisher’s Dynabeads Oligo (dT) was used to purify total RNA. The Illumina paired-end RNA-seq approach produced millions of 2 × 150 bp paired-end reads. However, only qualifying RNA samples were used for RNA-seq. Cutadapt-1.9 (https://cutadapt.readthedocs.io/en/stable/) (accessed on the 25 Novembe 2024) was used to filter low-quality traits reads. Sequence quality traits were then verified using FastQC 0.11.9 (https://sourceforge.net/projects/fastqc.mirror/files/v0.11.9/) (accessed on the 25 November 2024). Next, we used the HISAT2-2.2.1 (https://daehwankimlab.github.io/hisat2/) (accessed on the 25 November 2024) software program [20] to map all reads to the chicken reference genome. Two distinct groups were analyzed with the DESeq2 software (R4.1.2) [21,22]. DEGs were defined as genes with differential multiplicity FC ≥ 2 or FC ≤ 0.5 (|log_2_FC| ≥ 1) and a threshold criterion of q-value < 0.05.

### 2.6. Combined Analysis of Differential Genes and Differential Metabolites

The correlation between DEMs and DEGs was evaluated using the Pearson correlation coefficient. Afterward, the phenotype-key candidate gene-key metabolite interaction network was constructed using the OmicStudio tools (https://www.omicstudio.cn/tool/56, accessed on the 25 November 2024) software.

### 2.7. Statistical Analysis

We performed *t*-tests to determine significant differences between groups using Graph Prism software (v8.0.2), and data were expressed as mean ± SEM. *p* < 0.05 indicates a significant difference, and *p* < 0.01 indicates a highly significant difference (*, **, and *** stand for *p* < 0.05, *p* < 0.01, and *p* < 0.001, respectively). Images were generated by Graph Prism (v8.0.2) software.

## 3. Results

### 3.1. Meat Quality Traits Performance of Jingyuan Hens at Different Developmental Stages

**Figure 1:**
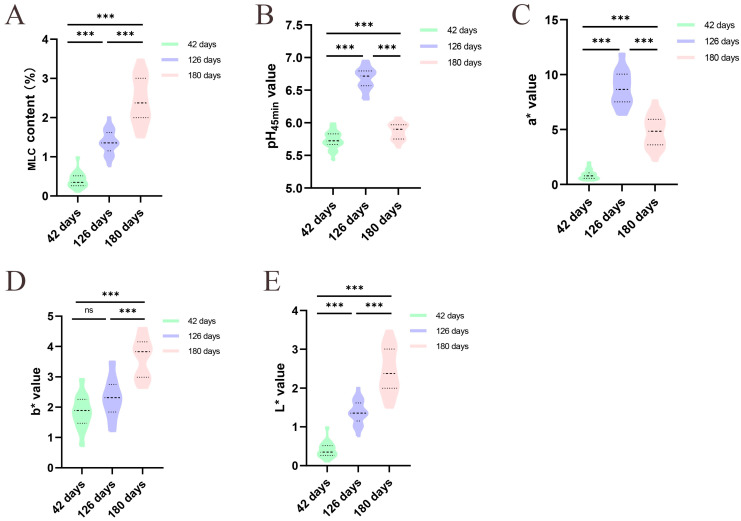
Meat quality traits performance of Jingyuan hens at different developmental stages, *n* = 30. (**A**) MLC; (**B**) pH_45min_ value; (**C**) meat color a* value; (**D**) meat color b* value; (**E**) meat color L* value. (ns and *** stand for *p* > 0.05 and *p* < 0.001, respectively).

We assessed the meat quality trait indices of the breast muscle tissues of Jingyuan hens that were 42-, 126-, and 180- days old. Age-related increases in MLC were highly significant (*p* < 0.001, Figure 1A). When compared to chickens that were 42- and 180- days old, the pH_45min_ value at 126-day-old was considerably higher (*p* < 0.001, Figure 1B). Furthermore, in comparison to the other two groups, the a* and L* values peaked at 126 days (*p* < 0.001, Figure 1C,E). Compared to those of the 42- and 126-day-old chickens, the b* values in 180-day-old chickens were significantly greater (*p* < 0.001, Figure 1D).

### 3.2. Metabolome QC and OPLS-DA Analysis

**Figure 2:**
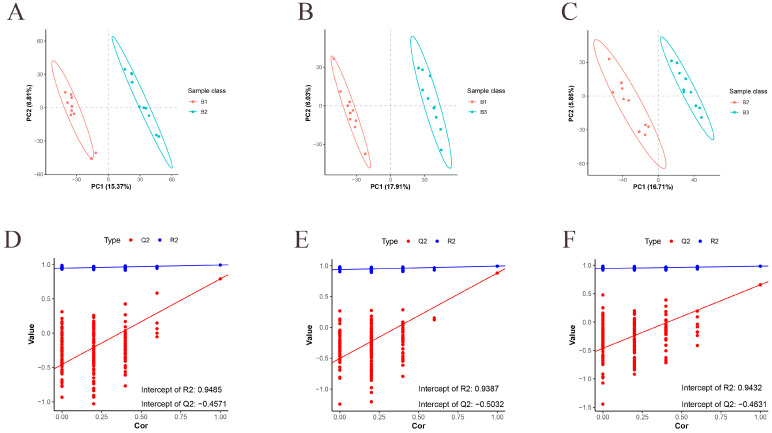
OPLS-DA analysis of metabolic groups. (**A**–**C**) OPLS-DA analysis for 42- and 126-day-old, 42- and 180-day-old, and 126- and 180-day-old chickens. (**D**–**F**) Replacement tests for 42- and 126-day-old, 42- and 180-day-old, and 126- and 180-day-old chickens. *n* = 10.

We first performed metabolomic sequencing analysis on three-day-old chicken breast muscle of Jingyuan chickens, and the overlap analysis of the total ion current maps of the samples in positive and negative ionization modes showed good reproducibility. The normalized clean metabolite table is shown in Table S2. The Pearson correlation coefficients of the quality traits control (QC) samples were close to 1, which proved the reproducibility and reliability of the data (Figure S1). The metabolites in the positive- and negative-ion modes were then subjected to further analysis. The correlation of all samples within and between groups was described using PCA (Figure S2). OPLS-DA used supervised patterns to find sample correlations between all pairs of groups. The comparison groups for 42- and 126-day-old, 42- and 180-day-old, and 126- and 180-day-old chickens demonstrated distinct group separation and strong sample clustering within the groups (Figure 2A–C). We used a permutation test to prevent overfitting. For any two groups, R^2^ values > 0.9 and Q2 values < 0.4 show that these models are not overfitted and are stable (Figure 2D–F).

### 3.3. Differential Metabolite Analysis

**Figure 3:**
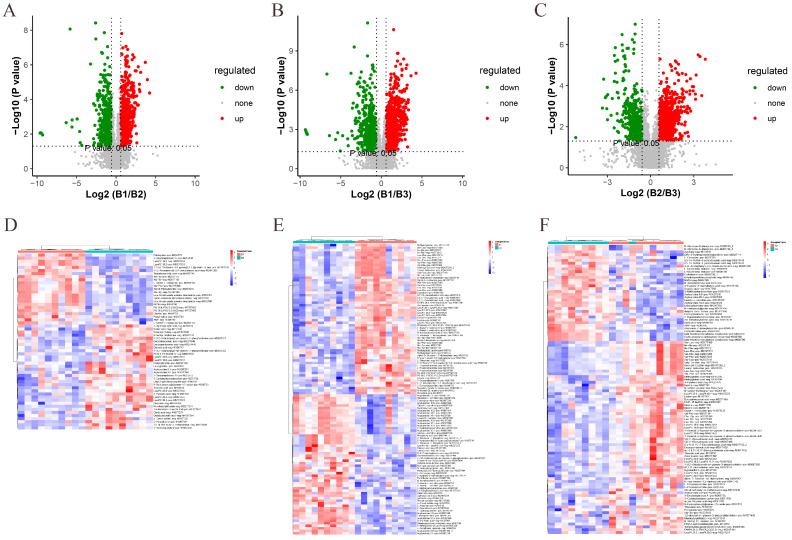
Screening and identification of DEMs between different age groups. (**A**) Volcano plots for 42- and 126-day-old, (**B**) 42- and 180-day-old, and (**C**) 126- and 180-day-old chickens. Each dot represents a DEM. Red dots represent significantly upregulated metabolites and green dots represent significantly downregulated metabolites. Gray dots represent metabolites that were not significantly different. Dot sizes indicate VIP values. (**D**) Hierarchical clustered heatmaps were generated for DEMs identified at 42 and 126 days, (**E**) 42 and 180 days, and (**F**) 126 and 180 days. Red color represents a positive correlation and blue color represents a negative correlation.

We performed a two-by-two analysis of the metabolites identified at three different ages. Based on the conditions of VIP > 1, *p* value < 0.05, FC ≥ 1.5, or FC ≤ 0.667, DEMs were screened between 42 and 126 days, 42 and 180 days, and 126 and 180 days. Volcano plots were used to illustrate the distribution of DEMs between different age groups. In the 42- and 126-day-old comparison, a total of 1390 DEMs were identified in both the positive- and negative-ion modes: 918 DEMs were upregulated, and 696 DEMs were downregulated (Figure 3A, Table S3). In the comparison of 42- and 180-day-old chickens, a total of 1726 DEMs were identified: 945 DEMs were upregulated, and 781 DEMs were downregulated (Figure 3B, Table S4). In the comparison of 126- and 180-day-old chickens, a total of 1527 DEMs were identified: 1008 DEMs were upregulated, and 519 DEMs were downregulated (Figure 3C, Table S5). Furthermore, a hierarchical clustered heatmap was created using the relative abundance of DEMs found in any two groups (Figure 3D,E).

### 3.4. Trend Analysis of Differential Metabolite Expression

**Figure 4:**
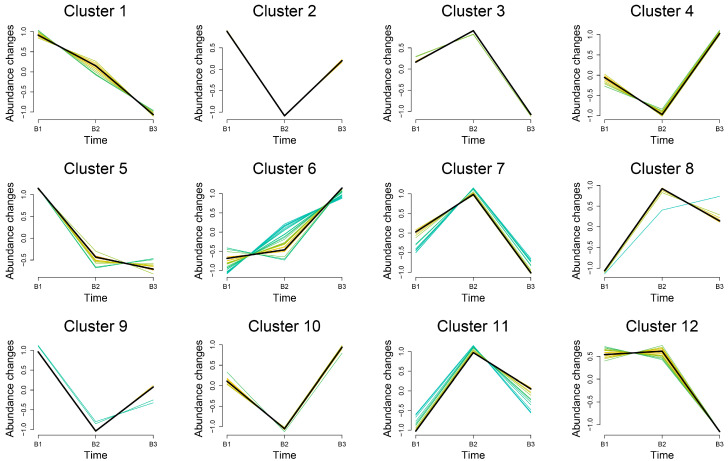
Trend analysis of differential metabolite expression in breast muscle in 3 age groups. A cluster may contain multiple functionally related genes, and these color lines represent these genes in different expression profiles, demonstrating their respective trajectories of expression changes.

The trends of DEMs in the breast muscles of the three age groups were subjected to Mfuzz clustering analysis. The abundance of 29 DEMs in cluster 6 exhibited an increasing trend with increasing age, while 10 DEMs in cluster 1 showed a substantial drop with increasing age, according to the results, which displayed 12 patterns (Figure 4). The metabolites grouped in cluster 1 and cluster 6 are displayed in Table 1 and Table 2, respectively. In both clusters, we discovered that fatty acyls were most prevalent. Cluster 6 contained eight fatty acids: 2-hydroxy-2-methylbutyric acid, Lauroyl-L-carnitine, Lauroylglutarylcarnitine, (2R)-3-Hydroxyisovaleroylcarnitine, Linoleoylcarnitine, Acylcarnitine 9:0, and Acylcarnitine 20:4. L-propionylcarnitine, methylglutaric acid, 5,8,11-eicosatrienoic acid, 8Z,11Z-eicosadienoic acid, 1,2,5,6-tetrahydro-4H-pyrrolo[3,2,1-ij]quinolin-4-one, and choline were the six fatty acyls that were clustered in cluster 1.

### 3.5. KEGG Pathway Enrichment Analysis

**Figure 5:**
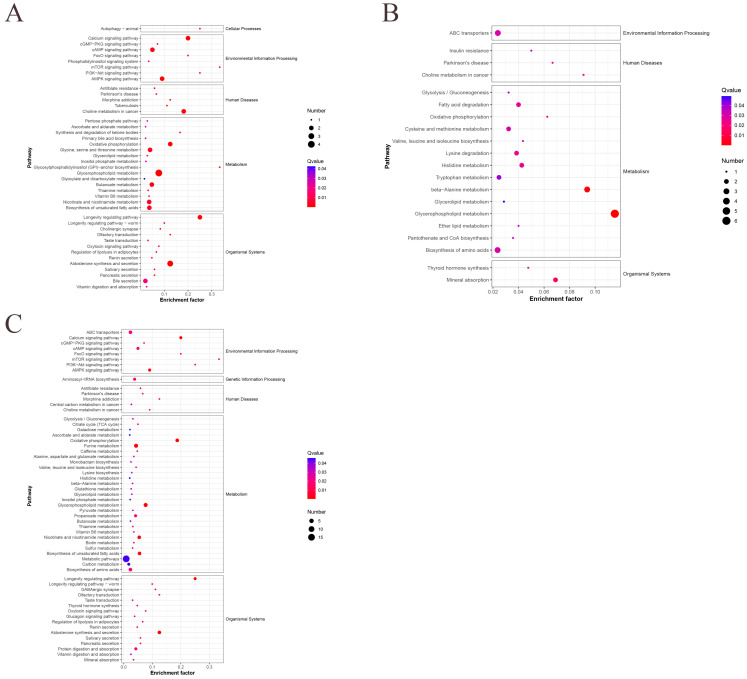
KEGG pathways significantly enriched in DEMs between different age groups. (**A**) KEGG pathways at 42 and 126 days old, (**B**) 42 and 180 days old, and (**C**) 126 and 180 days old.

**Figure 6:**
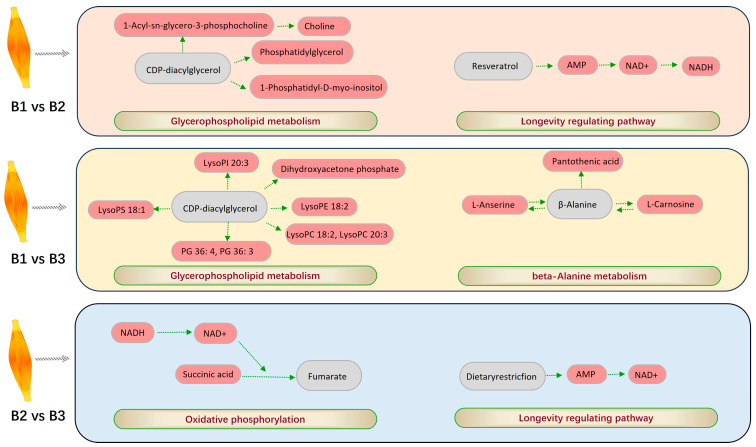
The most important pathways and enriched DEMs between different age groups.

The DEMs in the three age groups were then subjected to KEGG pathway enrichment analysis. In the 42- and 126-day-old, 42- and 180-day-old, and 126- and 180-day-old groups, a total of 51, 40, and 73 pathways were annotated to KEGG pathways; 43, 29, and 63 of these pathways were highly enriched, respectively (Table S6). The glycerophospholipid metabolism and longevity-regulating pathway was the most significantly enriched DEMs found in the 42- and 126-day-old group (Figure 5A). The two most significant pathways found in the 42- and 180-day-old group were beta-alanine metabolism and glycerophospholipid metabolism (Figure 5B). The two most significant pathways found in 126- and 180-day-old chickens were those that regulate lifespan and oxidative phosphorylation (Figure 5C). By filtering out the most important pathways in each comparison group, we further analyzed these pathways to identify DEMs that were significantly enriched in the most important pathways (Table 3). The most important pathways and DEMs were then visualized for each age group (Figure 6).

### 3.6. Joint Analysis of Transcriptome and Metabolome

**Figure 7:**
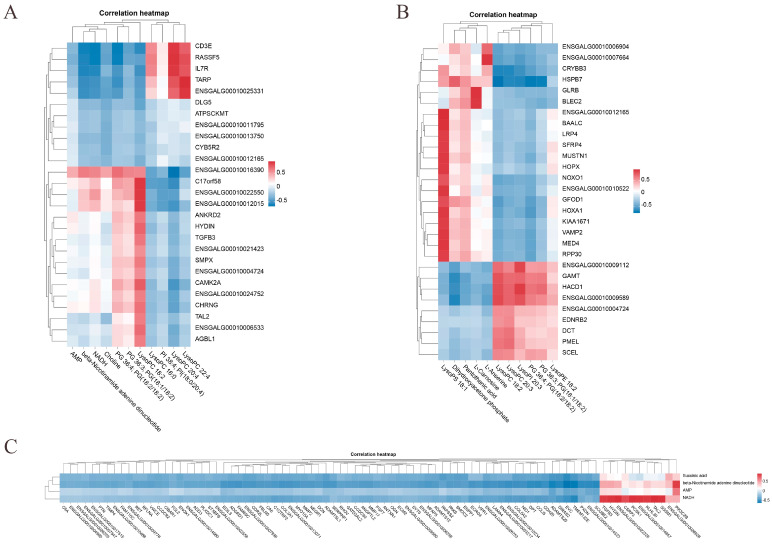
Integration analysis of DEMs and DEGs in the most important pathways between different age groups. (**A**) Heatmap of correlation between 42- and 126-day-old, (**B**) 42- and 180-day-old, and (**C**) 126- and 180-day-old chickens.

The correlation analysis of DEMs and DEGs across age groups was then carried out. For further examination, genes with absolute correlation coefficients of 0.9 or higher with DEMs in the most significant pathways were chosen. The DEGs that were most substantially linked with the DEMs in various age groups are displayed in Table 4, and correlation heatmaps were utilized to clarify the interactions between DEMs and DEGs in the most significant pathways (R >|0.7|, *p* < 0.01). In the 42- and 126-day-old groups, the glycerophospholipid metabolism pathway was linked to LysoPC 18:2, which was also highly significantly positively correlated with *CHRNG*, *ENSGALG00010006533*, *HYDIN*, *CAMK2A*, and *ENSGALG00010012015*, *ENGALG00010022550*, and *C17orf58*. *CD3E*, *IL7R*, *RASSF5*, *ENSGALG00010025331*, and *TARP* all showed highly significant positive correlations with LysoPC 22:4 and LysoPC 20:4. There was a highly substantial positive correlation between ENSGALG00010016390 and PG 36:4 and PG (18:2/18:2) (Figure 7A). In the 42- and 180-day-old groups, LysoPC 18:2, which is likewise abundant in the Glycerophospholipid metabolism pathway, had a significantly significant positive connection with *HACD1*, *GAMT*, *ENSGALG00010009112*, *ENSGALG00010009589*, and *HSPB7*. *GLRB* and *BLEC2* exhibited a significantly substantial positive connection with beta-L-carnosine in the alanine metabolism pathway. *ENSGALG00010007664* and *ENSGALG00010006904* had a significantly substantial positive connection with l-Anserine. An extremely substantial positive connection was seen between dihydroxyacetone phosphate and *HSPB7* (Figure 7B). In the 126- and 180-day-old groups, NADH enriched in the oxidative phosphorylation pathway was highly significantly positively correlated with *ENSGALG00010022328*, *TAL2*, *PCSK1*, *SGSM1*, *HYDIN*, *ENSGALG00010014657*, *KLHL30*, *TGFB3*, and *CSRP3*. HSPB7 and beta-nicotinic acid showed a favorable correlation (Figure 7B). association. A highly substantial positive connection was seen between beta-nicotinamide adenine dinucleotide and *PIK3C2B* (Figure 7C).

### 3.7. Identification of Meat Quality Traits Related Candidate Genes and Metabolites

**Figure 8:**
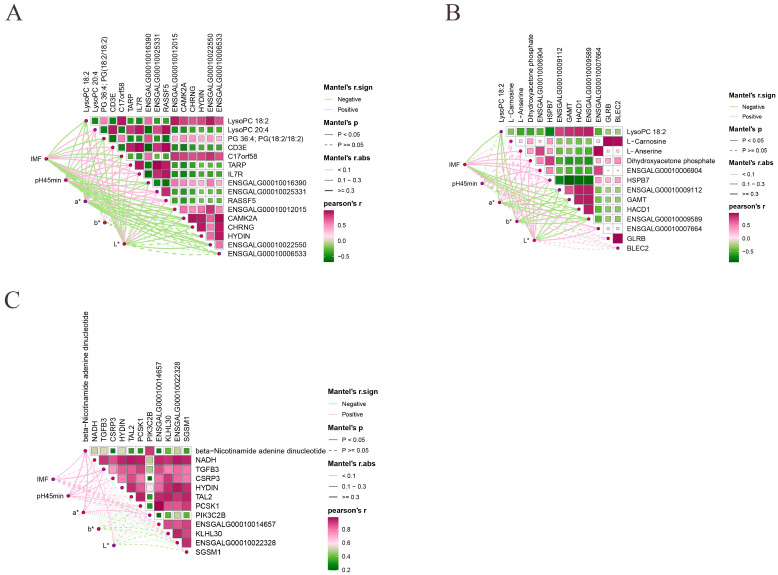
Pearson correlation analysis of key metabolites and genes with meat quality traits phenotypes. (**A**) 42- and 126-day-old, (**B**) 42- and 180-day-old, and (**C**) 126- and 180-day-old.

We conducted Pearson correlation analyses of key DEMs and DEGs in the most significant pathways with meat quality traits characteristics in order to identify the most pertinent candidate genes for meat quality traits performance in Jingyuan chickens. MLC, pH_45min_, a*, and L* showed significant positive correlations with LysoPC 20:4, *CD3E*, *TARP*, *IL7R*, *ENSGALG00010025331*, and *RASSF5*, while PG 36:4 showed significant negative correlations with LysoPC 18:2, PG (18:2/18:2), C17orf58, *ENSGALG00010016390*, *ENSGALG00010012015*, *CAMK2A*, *CHRNG*, and *ENSGALG00010022550* (Figure 8A). MLC, pH_45min_, a*, and L* showed significant and positive correlations with L-Anserine, Dihydroxyacetone phosphate, *ENSGALG00010006904*, and *HSPB7* in the 42- and 180-day-old groups, while *ENSGALG00010009112*, GAMT, HACD1, and ENSGALG00010009589 showed significant negative correlations with LysoPC 18:2 (Figure 8B). In the 126- and 180-day-old groups, there was a substantial and positive correlation between beta-Nicotinamide adenine dinucleotide and MLC, pH_45min_, a*, and L* (Figure 8C). Remarkably, neither of the two comparison groups showed a significant correlation between b* and important genes and metabolites.

## 4. Discussion

MLC controls the organoleptic properties of meat, including its flavor, tenderness, juiciness, and other characteristics, and is closely linked to meat quality traits [23]. Lipoprotein lipase facilitates the delivery of lipids to the breast muscle tissue, where they are digested to release fatty acids for oxidative energy or deposited to create MLC [24,25]. For example, the breast muscles of Korean groundhogs contain more crude fat as they age [26]. Similarly, our study found that the amount of MLC in the breast muscle of Jungyuan chickens increased as the chickens aged, specifically at 42, 126, and 180 days old. The color, tenderness, and water-holding capacity of chicken meat are all influenced by pH, a crucial quality traits metric that also serves as a key indicator of the freshness of meat products [27,28]. As animals reach more mature physiological stages, lactate production and accumulation may decrease, potentially due to shifts in muscle metabolism and changes in enzyme activity [29]. In chickens, some enzymes associated with glycolysis, such as lactate dehydrogenase, may change as they age, leading to decreased lactate production, which results in a higher pH [30,31]. At 126 days, Jingyuan chickens are at a critical period of sexual maturity, when their metabolism and enzyme activities are high. During this stage, lactate accumulation and acid release are low, which may result in a relatively high pH [32]. The color of chicken meat is determined by the pigments in the muscle, primarily myoglobin and fat [33]. As chickens age, physiological processes such as muscle growth, maturation, and fat deposition influence muscle color [34]. In our study, at 126 days, the myoglobin content in the muscle was higher, contributing to a reddish coloration of the meat and higher a* values. When the muscle became more oxidized, it resulted in a lighter color and higher L* values. As chickens reached 180 days of age, fat accumulation increased, and yellow fat deposits may have resulted in higher b* values.

Most significantly, the DEMs found in the 42- and 126-day-old comparison group were concentrated in pathways that upregulated longevity and glycerophospholipid metabolism. The two most significant metabolic pathways identified in the 42- and 180-day-old comparison group were enriched glycerophospholipid metabolism and beta-alanine metabolism. When comparing 126- and 180-day-old chickens, the two most significant pathways were those that upregulated the longevity-regulating pathway and oxidative phosphorylation. Glycerophospholipids, the most prevalent phospholipids in mammals, are composed of various substituent groups and phosphatidic acid [34,35]. Glycerophospholipid metabolism is likely crucial for intramuscular lipid synthesis, as it is associated with the conversion of fatty acids through phosphatidic acid synthesis [36]. Oxidative phosphorylation is an essential pathway for cellular energy metabolism, enabling mitochondria to produce ATP through redox reactions [37]. Within mitochondria, fatty acids undergo oxidation, generating reduced forms of nicotinamide adenine dinucleotide (NAD) and flavin adenine dinucleotide (FAD). These compounds are then involved in the longevity-regulating pathway, which generates a significant amount of ATP. Under normal conditions, when glucose is available, it is the primary substrate used to produce ATP in poultry breast muscles, fueling the oxidative phosphorylation/electron transport chain pathway. The resulting pyruvate from glucose metabolism is translocated into the mitochondria to support this process. Oxidative phosphorylation and the longevity-regulating pathway together provide the energy required for organismal activity, particularly in slow-growing strains, where breast muscle fibers are predominantly composed of Type I muscle fibers [38]. L-carnosine, produced via beta-alanine metabolism, contributes to the oxidation of β-oxidants to generate ATP, which increases energy production and is directly associated with the oxidation and consumption of fat [39]. In summary, these metabolic pathways play a significant role in the body’s energy supply and lipid metabolism, and their interactions may have a direct or indirect impact on MLC deposition.

In cluster 1, the abundance of 10 DEMs (6 fatty acyls) significantly declined with increasing age, whereas in cluster 6, the abundance of 29 DEMs (8 fatty acyls) tended to increase with age, according to our Mfuzz cluster analysis of the trends of DEMs in the breast muscles of the three age groups. Levels of oxidative stress are typically elevated in sedentary hens as they age. Oxidative stress affects cellular and metabolic pathways, potentially resulting in the slowing down or alteration of some metabolic pathways, which may lead to lower concentrations of relevant metabolites [40]. For example, the synthesis and secretion of bile acids (such as Taurodeoxycholic acid) may decrease with declining liver function, leading to a reduction in the abundance of related metabolites [41]. Additionally, the muscle and tissue utilization efficiency of amino acid metabolites (such as Prolyl-Valine and Threonine) decreases, and the rate of synthesis and metabolism slows down [42]. As age increases, alterations in fatty acid synthesis and metabolic pathways occur, resulting in a decrease in the abundance of fatty acid metabolites (such as 5,8,11-Eicosatrienoic acid, 8Z,11Z-Eicosadienoic acid, etc.) in the body [43]. L-Propionylcarnitine and Methylglutaric acid, intermediates associated with energy metabolism—especially fatty acid oxidation—also show decreased abundance as metabolic efficiency declines [44]. Consequently, the abundance of some metabolites may decrease. Eight fatty acyls were clustered in cluster 6, including seven carnitines and one butyric acid. Carnitine, as an intermediate metabolite of organic acids and fatty acids, serves as a carrier for the transport of long-chain fatty acids, facilitating their entry into the mitochondrial matrix for fatty acid β-oxidation metabolism [45,46]. Carnitine not only accelerates fatty acid metabolism during β-oxidation but also provides energy for the normal physiological activities of the organism [47,48]. In this study, we also found that most of the DEMs clustered in cluster 6, and the metabolic pathways they were enriched in were related to fatty acid metabolism, energy production, and fat storage. These processes exhibited varying degrees of activity during the growth stages of Jingyuan chickens. At 42 and 126 days old, chickens primarily rely on protein synthesis and growth for energy expenditure. However, with age, there is an increasing focus on energy storage and regulation of lipid metabolism, which may lead to an increase in the abundance of certain metabolites.

Studies integrating metabolomics and transcriptomics have focused on revealing how multiple genes and signaling pathways regulate and improve muscle meat quality traits [49]. Our results showed that LysoPC 20:4, LysoPC 18:2, PG 36:4, and PG (18:2/18:2) were associated with *CD3E, TARP, IL7R, ENSGALG00010025331, RASSF5,* and *C17orf58* at 42 and 126 days old. Additionally, *ENSGALG00010016390*, *ENSGALG00010012015, CAMK2A*, *CHRNG*, and *ENSGALG00010022550* were significantly correlated with and enriched in the glycerophospholipid metabolism pathway. LysoPCs can influence phospholipid metabolism [50,51]. Choline stimulates the expression of genes responsible for lipid transport, translocation, hydrolysis, and the biosynthesis of long-chain fatty acids in muscle, thereby promoting MLC deposition [52]. In this study, LysoPC 20:4, LysoPC 18:2, PG 36:4, and PG (18:2/18:2) were involved in fatty acid metabolism in vivo through the glycerophospholipid metabolism pathway, thereby affecting MLC deposition. LysoPC 18:2, which is also enriched in the glycerophospholipid metabolism pathway, was highly significantly positively correlated with *ENSGALG00010009589*, *HACD1*, *GAMT*, *HSPB7*, and *ENSGALG00010009112* in 42- and 180-day-old chickens. In the beta-alanine metabolism pathway, L-anserine showed a highly significant positive correlation with *ENSGALG00010007664* and *ENSGALG00010006904*. Dihydroxyacetone phosphate was significantly positively correlated with *HSPB7* and involved in lipid metabolism via beta-alanine metabolism, where triglyceride catabolism in adipocytes produces glycerol and free fatty acids during lipolysis [53,54]. All these results are consistent with the findings of the present study. In the beta-alanine metabolism pathway, L-carnosine was highly significantly positively correlated with *GLRB* and *BLEC2*. Previous studies have shown that dietary addition of L-carnosine promotes lipolysis, which affects fat deposition [44,45]. Additionally, oxidative phosphorylation is the most important pathway at 126- and 180-day-old, with the key metabolite beta-nicotinamide adenine dinucleotide being significantly associated with meat quality traits indicators. Overall, these enriched metabolites are involved in energy supply and lipid metabolism in the organism through various metabolic pathways, which further influence MLC deposition and utilization.

## 5. Conclusions

The dynamics of important genes and metabolites influencing meat performance in Jingyuan chickens at various stages of growth and development were examined in this study using HPLC-HRMS. In contrast with 180-day-old hens, which had higher MLC and b* values, 126-day-old hens exhibited higher pH_45min_·, a*, and L* values. A trend analysis of metabolites in 42-, 126- and 180-day-old chickens showed that as age increased, 10 DEMs decreased, while 29 DEMs increased. The most significant pathway at 42 and 126 days old, according to combined transcriptome analysis, was glycerophospholipid metabolism. This pathway contained important metabolites (LysoPC 20:4, LysoPC 18:2, PG 36:4, PG (18:2/18:2)) and genes that affect meat quality traits (*CD3E*, *TARP*, *IL7R*, *ENSGALG00010025331*, *RASSF5*, *C17orf58*, *ENSGALG00010016390*, *ENSGALG00010012015*, *CAMK2A*, *CHRNG*, *ENSGALG00010022550*). Key genes (*ENSGALG00010006904*, *HSPB7*, *ENSGALG00010009112*, *GAMT*, *HACD1*, *ENSGALG00010009589*) and key metabolites (L-Anserine, Dihydroxyacetone phosphate, and LysoPC 18:2) were significantly correlated with meat quality traits indices. Glycerophospholipid metabolism and beta-alanine metabolism were the most significant pathways at 42 and 180 days old. In contrast, oxidative phosphorylation was the most prominent pathway at 126 and 180 days old, with meat quality traits indices being substantially correlated with the main metabolite, beta-nicotinamide adenine dinucleotide. This study clarifies the dynamics of MLC in Jingyuan chicken breast muscle at various developmental phases, and its findings will offer guidance on enhancing the flavor of chicken meat.

## Figures and Tables

**Table 1:** Differential metabolites enriched into cluster 6.

Metabolites	Class	FC	VIP	*p* Value
Acylcarnitine 9:0	Fatty acyls	0.16	3.48	0.01
Caffeoylcholine		0.23	3.19	0.01
Acylcarnitine 20:4	Fatty acyls	0.22	2.66	0.01
Dihydroxyacetone phosphate	Organooxygen compounds	0.24	2.76	0.01
2,6-Dihydroxybenzoic acid	Benzene and substituted derivatives	0.35	2.47	0.01
2-Piperidinone	Piperidines	0.44	2.24	0.01
Gallic acid	Benzene and substituted derivatives	0.24	2.67	0.01
L-Glutathione, reduced	Carboxylic acids and derivatives	0.55	1.76	0.01
Linoleoylcarnitine	Fatty acyls	0.27	2.46	0.01
Acylcarnitine 18:3	Fatty acyls	0.20	2.84	0.01
N-Undecanoylglycine	Carboxylic acids and derivatives	0.48	1.61	0.01
4-Hydroxyquinoline	Quinolines and derivatives	0.18	2.85	0.01
3-Methylglutarylcarnitine	Fatty acyls	0.34	2.13	0.01
Adenosine 5′-monophosphate	Purine nucleotides	0.54	1.76	0.01
D-Mannose-6-phosphate	Organooxygen compounds	0.30	2.08	0.01
Lauroyl-L-carnitine	Fatty acyls	0.38	2.19	0.01
D-Mannose-6-phosphate	Organooxygen compounds	0.39	1.85	0.01
(2R)-3-Hydroxyisovaleroylcarnitine	Fatty acyls	0.62	1.66	0.01
(Z)-14-Methyl-6-pentadecenoic acid	Purine nucleosides	0.55	1.42	0.01
Clofentezine		0.63	1.70	0.01
N-Acetylhistamine	Carboxylic acids and derivatives	0.45	1.50	0.01
Eremopetasinorol	Alcohols and polyols	0.56	1.39	0.01
L-Carnosine	Peptidomimetics	0.58	1.77	0.01
2-Hydroxy-2-methylbutyric acid	Fatty acyls	0.52	1.53	0.01
2-Fluoromethamphetamine		0.39	1.96	0.01
Dehydro-beta-Ionone	Prenol lipids	0.62	1.12	0.01
Xanthine	Carboxylic acids and derivatives	0.61	1.56	0.01
D-2-Phosphoglyceric acid		0.14	2.05	0.01
Succinic acid	Prenol lipids	0.41	1.69	0.01

**Table 2:** Differential metabolites enriched into cluster 1.

Metabolites	Class	FC	VIP	*p* Value
Taurodeoxycholic acid	Carboxylic acids and derivatives	2.27	1.59	0.03
Choline	Fatty acyls	1.75	1.51	0.01
1,2,5,6-Tetrahydro-4H-pyrrolo [3,2,1-ij] quinolin-4-one	Fatty acyls	2.18	1.78	0.01
Prolyl-Valine	Purine nucleotides	1.63	1.62	0.01
Methylglutaric acid	Fatty acyls	2.24	2.06	0.01
5,8,11-Eicosatrienoic acid	Fatty acyls	2.12	2.23	0.01
8Z,11Z-eicosadienoic acid	Fatty acyls	2.04	2.07	0.01
Threonine	Carboxylic acids and derivatives	2.08	2.03	0.01
L-Propionylcarnitine	Fatty acyls	3.72	2.20	0.01
LysoPC 20:3	Glycerophospholipids	2.50	2.44	0.01

**Table 3:** Differential metabolites enriched by the most important pathways in the three age groups.

Groups	Kegg Pathway	Metabolites	FC	*p* Value	VIP	Regulate
42 and 126 days old	Glycerophospholipid metabolism (map00564)	LysoPC 16:0	0.57	0.02	1.34	down
		LysoPC 18:2	2.32	0.00	2.33	up
		LysoPC 20:4	0.49	0.00	2.29	down
		PG 36:4; PG(18:2/18:2)	1.86	0.00	2.08	up
		PG 36:3; PG(18:1/18:2)	1.61	0.00	1.73	up
		PI 38:4; PI(18:0/20:4)	0.56	0.04	1.12	down
		Choline	1.75	0.01	1.51	up
		LysoPC 22:4	0.47	0.01	1.73	down
	Longevity-regulating pathway (map04211)	AMP	1.78	0.04	1.44	up
		beta-Nicotinamide adenine dinucleotide	4.57	0.00	3.20	up
		NADH	4.39	0.00	3.09	up
42 and 180 days old	Glycerophospholipid metabolism (map00564)	Dihydroxyacetone phosphate	0.24	0.00	2.76	down
		LysoPS 18:1	0.41	0.02	1.40	down
		LysoPI 20:3	3.00	0.00	2.58	up
		PG 36:4; PG(18:2/18:2)	1.87	0.00	1.83	up
		PG 36:3; PG(18:1/18:2)	1.67	0.00	1.62	up
		LysoPE 18:2	1.57	0.02	1.66	up
		LysoPC 18:2	3.34	0.00	2.93	up
		LysoPC 20:3	2.50	0.00	2.44	up
	beta-Alanine metabolism (map00410)	L-Carnosine	0.53	0.03	1.24	down
		Pantothenic acid	0.64	0.02	1.12	down
		L-Anserine	0.63	0.00	1.35	down
126 and 180 days old	Oxidative phosphorylation (map00190)	Succinic acid	0.41	0.04	1.69	down
		beta-Nicotinamide adenine dinucleotide	0.29	0.00	2.62	down
		NADH	0.18	0.00	3.36	down
	Longevity-regulating pathway (map04212)	AMP	0.44	0.00	2.09	down
		beta-Nicotinamide adenine dinucleotide	0.29	0.00	2.62	down

**Table 4:** Differential metabolite most significantly associated with differential metabolites in different age groups.

Metabolite	Groups	Regulate	Kegg Pathway	Genes
LysoPC 18:2	42 and 126 days old	up	Glycerophospholipid metabolism	*CHRNG, ENSGALG00010006533, HYDIN, CAMK2A, ENSGALG00010012015, ENSGALG00010022550, C17orf58*
PG 36:4; PG(18:2/18:2)		up		*ENSGALG00010016390*
LysoPC 22:4				*CD3E, IL7R, RASSF5, ENSGALG00010025331, TARP*
LysoPC 20:4		down		*ENSGALG00010025331, TARP, IL7R, CD3E, RASSF5*
LysoPC 18:2	42 and 180 days old	up	Glycerophospholipid metabolism	*ENSGALG00010009589, HACD1, GAMT, HSPB7, ENSGALG00010009112*
L-Carnosine		down	beta-Alanine metabolism	*GLRB, BLEC2*
L-Anserine		down		*ENSGALG00010007664, ENSGALG00010006904*
Dihydroxyacetone phosphate		down		*HSPB7*
NADH	126 and 180 days old	down	Oxidative phosphorylation	*ENSGALG00010022328, TAL2, PCSK1, SGSM1, HYDIN, ENSGALG00010014657, KLHL30, TGFB3, CSRP3*
beta-Nicotinamide adenine dinucleotide		down		*PIK3C2B*

## Data Availability

The raw data collected have been archived in NCBI under accession numbers PRJNA898123 and GSE294245.

## Supplementary Materials

The following supporting information can be downloaded at https://www.mdpi.com/article/10.3390/ani15131938/s1, Figure S1: Metabolomic quality control. (A) Total ion current map TIC in positive ion mode; (B) total ion current map TIC in negative ion mode; (C) calculation of inter-sample correlation using ion peak intensities; Figure S2: PCA of the three comparison groups. (A) 42- and 126-day-old, (B) 42- and 180-day-old, and (C) 126- and 180-day-old chickens; Table S1: Sample number information; Table S2: Metabolome data for normalized clean metabolite tables; Table S3: Identification of differential metabolites in the breast muscle of 42- and 126-day-old (B1 vs. B2 groups) Jingyuan chicken hens; Table S4: Identification of differential metabolites in the breast muscle of 42- and 180-day-old (B1 vs. B3 groups) Jingyuan chicken hens; Table S5: Identification of differential metabolites in the breast muscle of 126- and 180-day-old (B2 vs. B3 groups) Jingyuan chicken hens; Table S6: KEGG pathways significantly enriched for differential metabolites in three age groups.

## Abbreviations

The following abbreviations are used in this manuscript:

MLC	Muscle lipid content.
DEMs	Directory of open access journals.
DEGs	Three-letter acronym.
OPLS-DA	Linear dichroism.
PCA	Principal component analysis.
HMDB	Human Metabolome Database.
FC	Fold change.
dT	Dynabeads Oligo.
QC	Quality traits control
