# Supplementary material for: Metabolomics and Transcriptomics Reveal Age-Dependent Development of Meat Quality Traits in Jingyuan Chicken
Source: Animals (Basel). 2025 Jul 1;15(13):1938. doi: 10.3390/ani15131938 (PMC12248559; doi:10.3390/ani15131938)
Supplement: Supplementary file 1 [file animals-15-01938-s001.zip › animals-3680393-supplementary/Supplementary File(s)/Table S2.pdf]

Table S2. Metabolome data for normalized clean metabolite tables.

Mode	All	MS2	HMDB	KEGG	Annotated
negative	5319	306	2284	2082	2772
positive	7764	335	4564	3688	5215

Note: Mode: the mode in which the mass spectrometer detects a substance, mainly pos (positive ion mode) and neg (negative ion mode). All: the number of substances extracted by the XCMS software. Annotated: the number of substances that are finally annotated by the mass spectrometry data of the first and second levels. MS2: Number of secondary identifications, which refers to the number of substances that can be matched to both the primary m/z of a database substance and the fragment ion (secondary) m/z of a database substance.
