# Supplementary figures and images for: Metabolomics and Transcriptomics Reveal Age-Dependent Development of Meat Quality Traits in Jingyuan Chicken
Source: Animals (Basel). 2025 Jul 1;15(13):1938. doi: 10.3390/ani15131938 (PMC12248559; doi:10.3390/ani15131938)

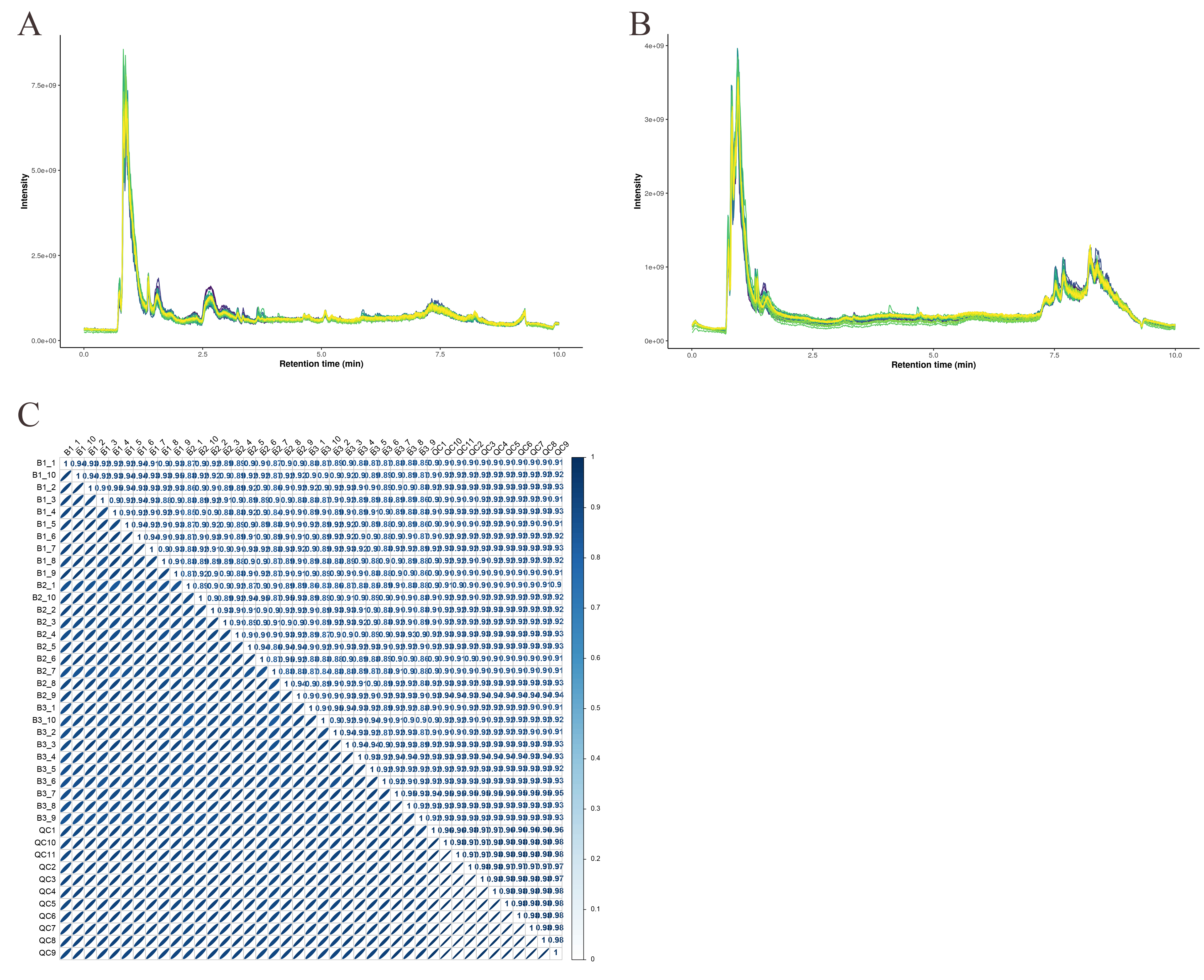

Supplement: Supplementary file 1 [file animals-15-01938-s001.zip › animals-3680393-supplementary/Supplementary File(s)/Figure S1.jpg]

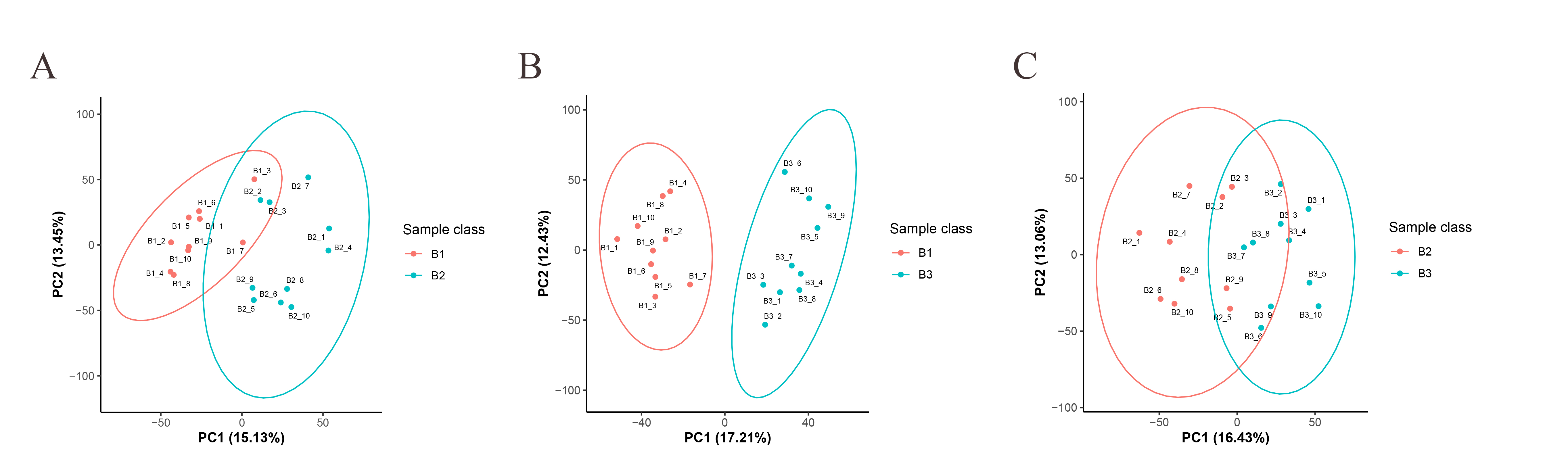

Supplement: Supplementary file 1 [file animals-15-01938-s001.zip › animals-3680393-supplementary/Supplementary File(s)/Figure S2.jpg]
